# How to Be an Informed Consumer of Evidence Ratings: It’s in the Details

**DOI:** 10.5888/pcd16.190067

**Published:** 2019-09-05

**Authors:** Alison Bergum, Lael Grigg, Marjory L. Givens, Bridget Booske Catlin, Julie Willems Van Dijk

**Affiliations:** 1 Population Health Institute, University of Wisconsin–Madison, Madison, Wisconsin; 2 Wisconsin Department of Health Services, Madison, Wisconsin

## Abstract

What are evidence-based strategies and how can public health practitioners find evidence without conducting extensive literature reviews? We developed an inventory of clearinghouses and other resources that disseminate research on evidence of effectiveness. We examined differences in evidence classification among 6 evidence clearinghouses that rate the effectiveness of community-level strategies to address determinants of health. Most evidence clearinghouses clearly defined their scope, but only a few clearinghouses explicitly defined the types of strategies they assess (eg, programs, policies, practices). The term “evidence-based” was widely used, but definitions and standards were inconsistent across organizations and disciplines. Evidence clearinghouses varied in the way they used evidence rating classifications and criteria for assigning ratings. Attention to detail is important. The criteria for the top rating of some evidence clearinghouses, for example, require a more thorough literature review with more robust results than the criteria for the top rating of others. In addition, some clearinghouses report only on strategies considered to be evidence-based, whereas others also report on strategies that have no effect, mixed evidence, or no qualifying studies, demonstrating that a listing of a strategy by an evidence clearinghouse does not necessarily mean that it is effective. We conclude by providing guidance for users of evidence clearinghouses about how to interpret and effectively apply rating criteria across platforms: look closely at the details of how clearinghouses assign their ratings and be aware of similarities and differences when you are aligning potential strategies with your local priorities. We encourage communities to balance evidence with local needs, resources, and culture in strategy selection and funding decisions.

**Summary**

**What is already known on this topic?**

Public health practitioners are increasingly aware of the importance of considering evidence about effectiveness when selecting strategies for implementation to improve community health.

**What is added by this report?**

This report offers an inventory of evidence clearinghouses for disseminating research on evidence and a review of the approaches used by a subset of these clearinghouses that provide summary ratings of evidence.

**What are the implications for public health practice?**

Understanding the types of strategies these clearinghouses review and how they develop their summary ratings is key knowledge for public health practitioners to make informed decisions about potential strategies for implementation.

**MEDSCAPE CME**

In support of improving patient care, this activity has been planned and implemented by Medscape, LLC and *Preventing Chronic Disease*. Medscape, LLC is jointly accredited by the Accreditation Council for Continuing Medical Education (ACCME), the Accreditation Council for Pharmacy Education (ACPE), and the American Nurses Credentialing Center (ANCC), to provide continuing education for the healthcare team.

Medscape, LLC designates this Journal-based CME activity for a maximum of 1.00 AMA PRA Category 1 Credit(s)™. Physicians should claim only the credit commensurate with the extent of their participation in the activity.

Successful completion of this CME activity, which includes participation in the evaluation component, enables the participant to earn up to 1.0 MOC points in the American Board of Internal Medicine’s (ABIM) Maintenance of Certification (MOC) program. Participants will earn MOC points equivalent to the amount of CME credits claimed for the activity. It is the CME activity provider’s responsibility to submit participant completion information to ACCME for the purpose of granting ABIM MOC credit.

Release date: September 5, 2019; Expiration date: September 5, 2020

**Learning Objectives**

Upon completion of this activity, participants will be able to:

1. Describe how evidence clearinghouses rate evidence, according to a report and review
2. Determine lessons learned from reviewing a sample of clearinghouses’ evidence of effectiveness ratings, according to a report and review
3. Identify guidance needed by public health practitioners, community leaders, and policy makers to be informed consumers of evidence clearinghouses that summarize evidence about health improvement efforts, according to a report and review

**EDITOR**

Ellen Taratus, MS

Editor

Preventing Chronic Disease

Disclosure: Ellen Taratus, MS, has disclosed no relevant financial relationships.

**CME AUTHOR**

Laurie Barclay, MD

Freelance writer and reviewer

Medscape, LLC

Disclosure: Laurie Barclay, MD, has disclosed no relevant financial relationships.

**AUTHORS**

Alison Bergum, MPA

Population Health Institute

University of Wisconsin–Madison

Disclosure: Alison Bergum, MPA, has disclosed no relevant financial relationships.

Lael Grigg, MPA

Population Health Institute

University of Wisconsin–Madison

Disclosure: Lael Grigg, MPA, has disclosed no relevant financial relationships.

Marjory L. Givens, PhD

Population Health Institute

University of Wisconsin–Madison

Disclosure: Marjory L. Givens, PhD, has disclosed no relevant financial relationships.

Bridget Booske Catlin, PhD

Population Health Institute

University of Wisconsin–Madison

Disclosure: Bridget Booske Catlin, PhD, has disclosed no relevant financial relationships.

Julie Willems Van Dijk, PhD

Wisconsin Department of Health Services

Madison, Wisconsin

Disclosure: Julie Willems Van Dijk, PhD, has disclosed no relevant financial relationships.

## What Is Evidence and Why Is It Important?

Since the early 1990s, evidence-based decision making has gained prominence in the field of medicine, followed by the field of public health. In medicine and public health, evidence typically refers to research evidence, rather than experiential or contextual evidence (1,2). Our study examines best available research evidence as both strength of evidence and effectiveness. “Strength of evidence” refers to how rigorously a program, policy, or practice has been evaluated and to the quality and quantity of evidence available to determine whether the program or policy is producing the desired outcomes. Effectiveness considers whether the outcomes observed are, in fact, a product of the program, policy, or practice itself and whether those outcomes are desirable or not desirable (2).

Systematic reviews of randomized control trials (RCTs) are widely recognized as the gold standard of intervention research. Such reviews follow an established process for searching, critically appraising, and summarizing results of research studies, accounting for all relevant qualifying studies and their results and establishing whether research findings are consistent and generalizable across populations and settings. Individual studies (for example, an RCT, a cohort study, a case-control study, a case series, and a case report) vary in strength of evidence. Sometimes, however, no study is available, and practitioners might turn to expert opinion (3,4). Researchers acknowledge that best evidence can exist in various forms (5), often in tandem with contextual factors such as clinical expertise, patient preference, and environmental and organizational context (6). Medical literature describes various methods for assessing evidence to support clinical practice recommendations, such as the Grading of Recommendations Assessment, Development and Evaluation (GRADE) system (7); however, these methods are primarily designed to evaluate clinical practice rather than community-based interventions.

Community leaders and practitioners have numerous approaches to finding evidence and varying criteria for considering evidence in decisions (8). Understanding the details of approaches to synthesizing and rating evidence can help practitioners harness best evidence to implement locally applicable, effective solutions. Using what has been successful through research, evidence can drive smart investments and support wise allocation of scarce dollars and other resources. And, knowing whether strategies exist to address local priorities can inform decisions about when to innovate and when to adopt strategies that have already been tested and shown to be effective. When strategies that support local priorities have strong evidence of effectiveness, practitioners have a solid starting place for action. When strategies that support local priorities do not have strong evidence of effectiveness or cannot be implemented with fidelity, which increases the likelihood of expected results (9), innovation using new untested strategies can be a better approach, especially when combined with evaluation.

## Where Can Communities Find Evidence?

Searching the scientific literature for RCTs or other studies is often not feasible for public health practitioners or community members, largely because of limited time and access to scientific literature (10). Evidence clearinghouses offer registries of strategies that communities can implement to address local priorities. Some evidence clearinghouses also review and assess evidence to rate strategies on the basis of the strength of the evidence of effectiveness. All aim to help guide local strategy selection or design decisions, but approaches differ. Our use of the term “evidence clearinghouse” refers to all clearinghouses that support this aim, incorporating a spectrum of methods and content areas.

We developed a comprehensive, but not exhaustive, inventory of evidence clearinghouses and other resources that summarize evidence on strategies that address the multiple determinants of health (Table 1). We focused on clearinghouses that regularly update content and make it available through searchable web-based platforms. We identified these clearinghouses through a general internet search and by using search terms such as “evidence ratings” and “research clearinghouses” and reviewing inventories compiled by groups such as the Results First Initiative (14), the Bridgespan Group (15), and the Corporation for National and Community Service (16). Some clearinghouses, such as healthevidence.org and Strengthening Families Evidence Review, focus on the quality of individual studies. Others, such as The Guide to Community Preventive Services (The Community Guide), conduct systematic reviews and provide a summary rating. Clearinghouses such as What Works for Health (WWFH) (the authors’ clearinghouse, part of the County Health Rankings & Roadmaps program) consider study quality and rate intervention effectiveness. Our inventory notes 21 clearinghouses that rate intervention effectiveness (Table 1).

**Table 1:** Clearinghouses and Other Resources That Summarize Evidence About Strategies That Address the Multiple Determinants of Health

Name	Scope	Type of Strategy Assessed	Rates Intervention Effectiveness	Initiator	Strategy Rating Information
Best Evidence Encyclopedia	Education	Programs	Yes	Johns Hopkins University School of Education. This group also operates Evidence for ESSA, which provides access to information on programs meeting the evidence standards of the Every Student Succeeds Act (ESSA).	http://www.bestevidence.org/aboutbee.htm
Conduent Healthy Communities Institute (HCI): HCI’s Promising Practices Database is publicly available on many sites	Public health (broad)	Policies and programs	Yes	HCI was started by a faculty member at University of California–Berkeley. It has been through a series of buyouts. Bought by Xerox in 2015 and then split off as part of Conduent.	https://healthycities.zendesk.com/hc/en-us/articles/220164127-Promising-Practices-ranking-methodology
Rural Health Information Hub	Rural health	Programs	Yes	Formerly the Rural Assistance Center, it is funded by the Federal Office of Rural Health Policy to be a national clearinghouse on rural health issues.	https://www.ruralhealthinfo.org/project-examples/criteria-evidence-base
Social Programs that Work^b^	Social programs	Programs	Yes	Transitioning from the Coalition for Evidence-Based Policy to the Laura and John Arnold Foundation. Updates *Top Tier Evidence* by the Coalition.	http://evidencebasedprograms.org/about-this-site
The Guide to Community Preventive Services^c^	Public health (broad)	Policies and programs	Yes	The Community Preventive Services Task Force	https://www.thecommunityguide.org/about/our-methodology
What Works for Health^b^	Public health (broad)	Policies and programs	Yes	University of Wisconsin Population Health Institute	http://www.countyhealthrankings.org/take-action-improve-health/what-works-health/our-methods
Blueprints for Healthy Youth Development^a^^,^ b	Youth development	Programs	Yes	University of Colorado–Boulder Institute of Behavioral Science, in partnership with Annie E. Casey Foundation.	http://www.blueprintsprograms.com/criteria
California Evidence-Based Clearinghouse for Child Welfare^a^^,^ b	Child welfare	Programs	Yes	Funded by California Department of Social Services’ Office of Child Abuse Prevention. Work conducted by Rady Children’s Hospital–San Diego.	http://www.cebc4cw.org/ratings/scientific-rating-scale
Center for Evidence-Based Crime Policy	Community safety (policing)	Policies and programs	Yes	George Mason University Department of Criminology, Law, and Society	http://cebcp.org/evidence-based-policing/the-matrix/inclusion-criteria-methods-key
Change Library	Public health (broad)	Programs and policies	Yes	Change Library was adapted from the North Carolina Improvement Map (IMAPP), which was developed by Population Health Improvement Partners, US Department of Health and Human Services, and the Institute for Healthcare Improvement. The redesign of IMAPP to Change Library was funded by the Robert Wood Johnson Foundation, Illinois Public Health Institute, and members of 100 Million Healthier Lives.	https://www.100mlives.org/wp-content/uploads/2018/08/v4_Bright-Spot-Guide-8-15-18.pdf
Clearinghouse for Military Family Readiness^c^	Family and mental health issues	Programs and practices	Yes	Partnership funded by the US Department of Defense between the Office of the Deputy Assistant Secretary of Defense for Military Community and Family Policy and the US Department of Agriculture’s National Institute of Food and Agriculture through a grant/cooperative agreement with Penn State University.	Strategy rating categories include effective (RCT), effective (quasi-experimental), promising, unclear+, unclear, unclear-, ineffective. No definitions for these ratings were found.
CrimeSolutions.gov^a^^,^ b ^,^ c	Criminal justice	Policies and programs	Yes	US Department of Justice, Office of Justice Programs	https://www.crimesolutions.gov/about_evidencecontinuum.aspx
Home Visiting Evidence of Effectiveness^c^	Home visiting	Programs	Yes	US Department of Health and Human Services, contract with Mathematica Policy Research	https://homvee.acf.hhs.gov/Review-Process/4/-abbr-Department-of-Health-and-Human-Services-DHHS-abbr-Criteria-for-Evidence-Based-Program-Models/19/6
Innovation Station: Sharing Best Practices in Maternal & Child Health	Maternal and child health	Programs	Yes	Association of Maternal & Child Health Programs	http://www.amchp.org/programsandtopics/BestPractices/InnovationStation/Pages/Best-Practices-Program.aspx
Office of Juvenile Justice Delinquency Prevention-Model program guide^c^	Juvenile justice and delinquency	Programs	Yes	Office of Juvenile Justice and Delinquency Prevention	Uses CrimeSolutions.gov procedures https://www.ojjdp.gov/mpg/Home/About#mpg
What Works Clearinghouse Intervention reports^a^^,^ b ^,^ c	Education	Programs, (and policies, products and practices per site)	Yes	Managed by Institute of Education Sciences for US Department of Education, work conducted by various contracted organizations.	Criteria for rating individual studies: https://ies.ed.gov/ncee/wwc/Docs/referenceresources/wwc_info_rates_061015.pdf. Intervention effectiveness rating: https://ies.ed.gov/ncee/wwc/Docs/referenceresources/wwc_info_reporting_061015.pdf
What Works in Reentry Clearinghouse^a^^,^ b ^,^ c	Criminal justice (reentry)	Programs	Yes	Justice Center, The Council of State Governments	Draws from CrimeSolutions.gov
Agency for Healthcare Research and Quality Innovations Exchange	Health care	Programs	Yes	Agency for Healthcare Research and Quality	https://innovations.ahrq.gov/help/evidence-rating
National Registry of Evidence-based Programs and Practices^a^^,^ b ^,^ c	Substance abuse and mental health	Programs	Yes	Substance Abuse and Mental Health Services Administration (funding ended January 2018).	Strategy rating URL link is no longer available
Promising Practices Network^a^	Child well-being and welfare	Programs	Yes	RAND Corporation (archived in 2014).	http://www.promisingpractices.net/criteria.asp
Teen Pregnancy Prevention Evidence Review^a^^,^ b	Teen pregnancy	Programs	Yes	US Department of Health and Human Services, Department of Adolescent Health; contract with Mathematica Policy Research.	https://tppevidencereview.aspe.hhs.gov/pdfs/TPPER_Review%20Protocol_v5.pdf (pages 9–10)
Clearinghouse for Labor Evaluation and Research^c^	Labor and employment	Policies and programs	No	US Department of Labor	Rates the quality of individual studies, not strategies
healthevidence.org	Public health (broad)	Policies and programs	No	McMaster University	Rates the quality of systematic reviews, not strategies
Health Systems Evidence	Public health (broad)	Policies and programs	No	McMaster University	No ratings found on website
Public Health Law Review Evidence	Public health (broad)	Policies	No	National program of the Robert Wood Johnson Foundation; housed at Temple University Beasley School of Law.	No ratings found on website
Self-Sufficiency Research Clearinghouse^c^	Social and economic factors	Policies and programs	No	Sponsored by Office of Planning, Research and Evaluation, US Department of Health and Human Services; various contributors.	No ratings found on website
Strengthening Families Evidence Review^c^	Children and families	Programs	No	Office of Planning, Research, US Department of Health and Human Services, contract with Mathematica Policy Research.	Rates the quality of individual studies, not strategies
The Campbell Collaboration Library of Systematic Reviews	Social and economic factors	Policies	No	International research network	No ratings found on website
The Cochrane Library	Public health and health care	Policies and programs	No	International research network	No ratings found on website
Violence Prevention Evidence Base and Resources	Community safety	Policies and programs	No	Centre for Public Health, Liverpool John Moores University in collaboration with World Health Organization and US Centers for Disease Control and Prevention	Rates the quality of individual studies not strategies
What Works/LINKS database	Children and families	Programs	No	Child Trends	No ratings found on website
Youth Power — What Works	Children	Policies and programs	No	US Agency for International Development	No ratings found on website

a Included in the *Results First Clearinghouse Database User Guide* (11).

b Included in Results First Clearinghouse Database (12), as of November 2018.

c Included in list of evidence-based program directories on Youth.gov (13).

Each clearinghouse has its own scope of interest, methods, and rating classifications. Many clearinghouses also provide additional content to accompany evidence ratings and support effective decision making. Some, such as The Community Guide, WWFH, and Social Programs that Work (SPTW), provide cost-related information. This information ranges from The Community Guide’s economic effectiveness analysis (conducted for strategies they rate as “recommended”) to study details noted by WWFH and SPTW. Some also emphasize tools or content that can bolster efforts to increase equity or reduce disparities in health-related outcomes. WWFH, for example, assesses the likely effect of each strategy among socioeconomic, racial/ethnic, and geographic groups. Many clearinghouses that assess and rate evidence also provide examples, stories, or other action-focused resources to support implementation.

## How Is Evidence Rated?

To understand how evidence clearinghouses rate evidence, we selected a sample of clearinghouses that provide evidence of effectiveness ratings for strategies that affect multiple determinants of health. Multiple determinants of health are defined in several ways, for example, “genetics, behavior, social circumstances, environmental and physical influences, and medical care” (17). The County Health Rankings model (18), on which WWFH is based, and this analysis, exclude genetics. In selecting our sample, we excluded clearinghouses that rate the quality of individual studies about an intervention but do not assess the effectiveness of that intervention overall. We also excluded clearinghouses that indicated their content is no longer updated. We minimized the inclusion of clearinghouses that are part of the Results First Clearinghouse Database, “an online resource that brings together information on the effectiveness of social policy programs from nine national clearinghouses” (12), because Results First provides tables to help users compare and contrast these ratings (11).

Our focused review examined the work of the following 6 evidence clearinghouses: Best Evidence Encyclopedia (BEE); The Community Guide; Healthy Communities Institute (HCI); Rural Health Information Hub (RHIhub); SPTW (formerly the Coalition for Evidence-Based Policy); and WWFH.

We conducted a qualitative analysis of the scope, methods, and ratings as described on the website of each of the 6 selected clearinghouses, with particular attention to the literature assessment (eg, literature review, systematic review), the criteria used to assess the quality of individual studies, and the type and number of studies required to establish each rating. We also considered scope of interest and the types of strategies assessed. We completed reviews in September 2018 and confirmed our information in October 2018. We invited staff members from each of the 6 clearinghouses to provide feedback on the accuracy of our information.

Each evidence clearinghouse has its own scope of interest (Table 2). The types of strategies (eg, programs, policies) assessed also varies, and selection of these strategies is largely tied to scope of interest and approach to compiling and assessing the literature. BEE, SPTW, and WWFH monitor topic-relevant research to identify potential strategies for assessment; SPTW and WWFH consult with experts. The Community Guide has a set process and priority-setting criteria to determine which strategies will be assessed. HCI accepts submissions and reviews them for inclusion on all community sites; local site administrators can decide whether to include submissions that are not selected for inclusion on all HCI sites. RHIhub also accepts submissions and includes programs that address rural health issues, are implemented in a rural US community, and include a program contact.

**Table 2:** Rating Approaches of Selected Evidence Clearinghouses^a^

Characteristic	Best Evidence Encyclopedia	The Guide to Community Preventive Services	Healthy Communities Institute	Rural Health Information Hub	Social Programs That Work	What Works for Health
Scope of interest	Education	Multiple determinants of health, selected diseases and injuries	Multiple determinants of health	Rural health	Social issues	Multiple determinants of health
Type of strategies assessed	Programs	Policies, systems, and environmental change	Programs	Programs and interventions	Programs	Policies, systems and environmental change; some programs
Approach to compiling and assessing literature	Conducts systematic reviews with meta-analysis	Conducts systematic reviews	Seeks and accepts submissions	Seeks and accepts submissions	Conducts systematic reviews	Conducts comprehensive literature reviews
Study types considered^b^	RCTs and strong quasi-experimental designs	RCTs, QEDs, and some weaker study designs	Peer-reviewed studies	Peer-reviewed studies	RCTs and strong QEDs	RCTs, QEDs, and some weaker study designs
Replicability^b^	Systematic reviews of strong studies required for highest evidence rating	Conducts applicability assessment for highest evidence rating	Does not appear to require demonstration of replication	Requires successful implementation in more than one community via peer-reviewed program evaluations	Systematic reviews of strong studies required for highest evidence rating	Multiple strong studies, strong study implemented in multiple sites, or systematic reviews of strong studies required for highest evidence rating
Rating categories^b^	Strong; Moderate; Limited: strong evidence of modest effects; Limited: weak evidence of modest effects; No qualifying studies	Recommended; Insufficient evidence; Recommended against	Evidence-based practice; Effective practice; Good idea	Evidence-based; Effective; Promising; Emerging	Top tier; Near top tier; Suggestive tier	Scientifically supported; Some evidence; Expert opinion; Insufficient evidence; Mixed evidence; Evidence of ineffectiveness

Abbreviations: QED, quasi-experimental design; RCT, randomized controlled trial.

a Selected clearinghouses rate effectiveness of interventions that work within the multiple determinants of health and update content regularly; efforts were made to minimize overlap with existing analyses in this field. For details, see How is Evidence Rated section.

b See Table 3 for details, criteria vary by rating category.

**Scope of interest and type of strategies assessed.** WWFH, HCI, and The Community Guide address multiple determinants of health; the latter two also address several diseases and injuries. RHIhub focuses on programs and interventions in rural communities. SPTW focuses on social programs and BEE on education programs. Some, such as The Community Guide and WWFH, emphasize broadly defined policy, systems, and environmental change (PSE) strategies; WWFH also includes some named programs, such as Nurse Family Partnership and Reach Out and Read. Other clearinghouses, such as SPTW and HCI, focus more heavily on named programs.

**Approach to compiling and assessing literature.** The websites of these 6 clearinghouses indicate various approaches to compiling and assessing available literature in support of their evidence ratings. The Community Guide and SPTW conduct systematic reviews, and BEE conducts systematic reviews with meta-analysis. WWFH conducts an extensive literature review, informed by the principles of systematic review methods, to capture and assess available evidence in a shorter time frame than systematic reviews, allowing inclusion of more strategies than the aforementioned clearinghouses. HCI and RHIhub seek and accept submissions from evaluators, practitioners, and others, fostering dissemination of early practice-based results. Review criteria for submissions were not apparent in our search of these 2 websites; it was also unclear whether a formal literature review process is used to inform evidence ratings.

**Study types considered.** Studies vary in their ability to determine causality; reviewed clearinghouses vary in the types of studies required to support evidence rating assignments. SPTW and BEE include RCTs and strong quasi-experimental designs (QEDs) as the foundation for their rating assignments. The Community Guide and WWFH include RCTs, QEDs, and some weaker study designs in their reviews. Strong QEDs are based on sound theory, use comparison groups, and typically include multiple measurement points; weaker study designs are also based on sound theory but do not have comparison groups and might not include multiple measurement points (2). Although HCI and RHIhub require peer-reviewed studies, overall, these clearinghouses do not specify the types of studies required for each rating. HCI and RHIhub describe pre–post designs for their highest rating categories and appear to assign this rating to strategies studied with or without comparison groups.

**Replicability.** The 6 clearinghouses also vary in their approach to replication, or demonstrations of generalizability, which is important to ensure a study’s results are valid in different settings, with different populations, or at different times (2). BEE, SPTW, and WWFH require multiple strong studies, a strong study implemented in multiple sites, or systematic review(s) of strong studies for their highest evidence ratings. The Community Guide conducts an applicability assessment process to evaluate generalizability along with the criteria used to assign their highest evidence rating. RHIhub requires successful implementation in more than 1 community via peer-reviewed program evaluations as a means to gauge replicability. HCI does not appear to require a demonstration of replication; its highest rating category can be assigned on the basis of 1 study that demonstrates program success in 1 or more locations.

**Rating categories.** Each of the 6 clearinghouses has a unique scale for rating evidence and a unique number of ratings (Table 3). Most ratings indicate degree of effectiveness, and some ratings indicate additional evidence is needed. Most rating categories are favorable (eg, “strong,” “recommended, “effective”), but WWFH and The Community Guide also assign unfavorable ratings: WWFH assigns “evidence of ineffectiveness,” and The Community Guide assigns “recommended against.” WWFH is the only organization with the rating “expert opinion.” “Expert opinion” is assigned to new strategies or innovations that have limited or no qualifying research but are recommended by credible, impartial experts. Additionally, this category may be indicated for strategies with benefits that are not described in empirical literature (eg, adding a dental clinic in a rural area without dental providers improves access to oral health care for at least some residents) or are difficult to test. RCTs are not always practical, as clearly pointed out by Smith and Pell in their systematic review of studies examining parachute use (19). WWFH also differentiates between “mixed evidence” (when strategies have been tested more than once in strong studies and results are inconsistent) and “insufficient evidence” (when too few studies assess the strategy of interest), whereas other clearinghouses might not; for example, The Community Guide covers both categories under “insufficient evidence.”

**Table 3:** Rating Categories at 6 Selected Evidence Clearinghouses^a^

Evidence Clearinghouse	Rating Category	Rating Description^a^
Best Evidence Encyclopedia	Strong	At least 1 large randomized or randomized quasi-experimental study and 1 additional large qualifying study, or multiple smaller studies, with a combined sample size of 500 and an overall weighted mean effect size of at least +0.20.
	Moderate	Two large matched studies, or multiple smaller studies with a collective sample size of 500 students, with a weighted mean effect size of at least +0.20.
	Limited: strong evidence of modest effects	Studies meet criteria for moderate evidence of effectiveness, except that the weighted mean effect size is +0.10 to +0.19.
	Limited: weak evidence of modest effects	A weighted mean effect size of at least +0.20 based on ≥1 qualifying studies insufficient in number or sample size to meet the criteria for moderate evidence of effectiveness.
	No qualifying studies	No studies met inclusion standards.
The Guide to Community Preventive Services	Recommended	Systematic review of available studies provides strong or sufficient evidence that the intervention is effective. Categories of “strong” and “sufficient” evidence reflect the degree of confidence the Community Preventive Services Task Force (CPSTF) has that an intervention has beneficial effects. Categories do not directly relate to expected magnitude of benefits. Categorizations are based on several factors, such as study design, number of studies, and consistency of effect across studies.
	Insufficient evidence	Available studies do not provide sufficient evidence to determine if the intervention is effective or not. This does not mean that the intervention does not work but that additional research is needed to determine if the intervention is effective. Findings might include a rationale statement for CPSTF recommendation or other conclusions.
	Recommended against	The systematic review of available studies provides strong or sufficient evidence that the intervention is harmful or not effective.
Healthy Communities Institute	Evidence-based practice	At minimum, the program description includes information on the sponsoring organization, program goals, program implementation steps, and outcomes that demonstrated success in achieving the program goal in one or more localities. Results from an evaluation of the program include quantitative measures showing improvement in the outcome of interest after the implementation of the program (eg, increase in smoking cessation, not just the delivery of a smoking cessation program). The outcome measure is compared at relevant periods before and after the intervention or program implementation. Alternatively, the evaluation study compares the outcome between an intervention group and an appropriate control group. The study is of peer-review quality and presents data in a scientific manner; measurements of precision and reliability are included (eg, confidence intervals, standard errors), results from statistical tests show a significant difference or change in the outcome measure and relevant point estimates and *P* values. If results from an evaluation of a program are presented in a scientific manner and the outcome measure improved from baseline or in the control group but the difference was not significant, the practice is classified as effective and not evidence-based.
	Effective practice	At minimum, the program description includes information on the sponsoring organization, program goals, program implementation steps, and outcomes that demonstrated program success and/or promise in achieving the program goal in one or more localities. The results from an evaluation of the program include quantitative measures of improvement in outcome of interest (ie, increase in voter registration, not just delivery of voter registration drive) and/or the outcome measure increased or improved from baseline or in the control group but the difference was not significant.
	Good idea	The program description includes information on the sponsoring organization, program goals, program funding source, program implementation steps, and outcomes. The program evaluation is limited to descriptive measure(s) of success/accomplishment (eg, program participation rates, number of services/education sessions/radio messages provided). Programs that have not yet been evaluated, but which show promise in improving health or quality of life, are classified as Good Ideas until an evaluation is conducted. These programs are often newly implemented, and a program evaluation has not yet been conducted.
Rural Health Information Hub	Evidence-based	A review study of the approach in a peer-reviewed publication. Approach tried in more than one location or setting. Overall results were positive for the approach and may have varied by setting or location.
	Effective	Reported in a peer-reviewed publication. May include a single location or setting or multiple locations or settings. Reported results were positive.
	Promising	A formal program evaluation was conducted and results are available publicly or the results were confirmed by Rural Health Information Hub staff members and are available on request from the program contact. Typically includes a single location or setting. Program evaluation shows positive results.
	Emerging	Anecdotal account of a program, without documentation of a formal evaluation. Typically includes a single location or setting. Program result may be positive (success story), negative (lesson learned), or mixed.
Social Programs that Work	Top tier	Programs shown in well-conducted RCTs, carried out in typical community settings, to produce sizable, sustained effects on important outcomes. Top Tier evidence includes a requirement for replication: the demonstration of such effects in ≥2 RCTs conducted in different implementation sites, or, alternatively, in 1 large multi-site RCT. Such evidence provides confidence that the program would produce important effects if implemented faithfully in settings and populations similar to those in the original studies.
	Near top tier	Programs that meet almost all elements of the top tier standard and that need only 1 additional step to qualify. This category primarily includes programs that meet all elements of the top tier standard in a single study site but need a replication RCT to confirm the initial findings and establish that they generalize to other sites. This standard is best viewed as tentative evidence that the program would produce important effects if implemented faithfully in settings and populations similar to those in the original study.
	Suggestive tier	Programs that have been evaluated in ≥1 well-conducted RCTs (or studies that closely approximate random assignment) and found to produce sizable positive effects, but whose evidence is limited by only short-term follow-up, effects that fall short of statistical significance, or other factors. Such evidence suggests that the program may be an especially strong candidate for further research but does not yet provide confidence that the program would produce important effects if implemented in new settings.
What Works for Health	Scientifically supported	Strategies with this rating are most likely to make a difference. These strategies have been tested in multiple robust studies with consistently favorable results.
	Some evidence	Strategies with this rating are likely to work, but further research is needed to confirm effects. These strategies have been tested more than once and results trend favorable overall.
	Expert opinion	Strategies with this rating are recommended by credible, impartial experts but have limited research documenting effects; further research, often with stronger designs, is needed to confirm effects.
	Insufficient evidence	Strategies with this rating have limited research documenting effects. These strategies need further research, often with stronger designs, to confirm effects.
	Mixed evidence	Strategies with this rating have been tested more than once and results are inconsistent; further research is needed to confirm effects.
	Evidence of ineffectiveness	Strategies with this rating are not good investments. These strategies have been tested in multiple studies with consistently unfavorable or harmful results.

a Rating descriptions are from each evidence clearinghouse’s website.

## Key Lessons in Considering Evidence of Effectiveness Ratings Provided by Evidence Clearinghouses

**Look for information about the scope of interest and types of strategies included.** Most evidence clearinghouses in our review clearly define their scope of interest and outline a framework for the topics covered. However, the types of strategies assessed (eg, policy, program) might not be so well defined. Understanding the scope and types of strategies covered can help users search appropriately for strategies to address local priorities.

**Ascertain what constitutes “evidence-based” for each clearinghouse, because no consensus exists.** Among the clearinghouses we examined, there is no universal definition of “evidence-based.” Clearinghouses vary in the terminology they use to describe levels of evidence and effectiveness and the criteria used to assign their ratings. Although evidence clearinghouses provide a streamlined way to learn about evidence, it is important for practitioners to pay attention to how each clearinghouse defines each term used in their rating classifications.

**Understand that evidence clearinghouses weight research designs differently.** Some, but not all, clearinghouses give greater weight to evidence from systematic reviews, RCTs, and strong QEDs than to other study types, particularly in their highest evidence rating categories. Systematic reviews and RCTs are recognized as the gold standard of effectiveness; seeking out interventions with this level of evidence can be important when a community is scaling up an intervention or investing substantial time or money, or when political stakes for success are high.

**Recognize differences in evidence clearinghouses’ requirements for literature review and their considerations of study quality and quantity.** Some clearinghouses search for evidence more systematically and judge study quality and design more strictly than others. Some also emphasize replicability more heavily. Yet others focus more on dissemination of early practice-based results. Understanding the breadth and replicability of studies provides practitioners with critical information as they consider deploying interventions in their own community.

**Be aware that most evidence clearinghouses do not assign ratings for ineffectiveness, expert opinion, or mixed results.** Only 2 clearinghouses that we examined closely include information about strategies with evidence of ineffectiveness, and WWFH is the only one that has the category “expert opinion.” Exploring evidence along the entire continuum of effectiveness can provide practitioners with information about ineffective policies or programs that might need to end, strategies with mixed evidence that may need a closer look, and strategies rated “insufficient evidence” or “expert opinion” that may especially benefit from more rigorous evaluation designs.

In general, more focus appears to be on what works rather than on what does not or is unknown. This discrepancy is likely due, at least in part, to the fact that more literature is available for what works than what does not — partially a result of publication bias (20). This focus on what works raises 2 important caveats. First, inclusion of a strategy in an evidence clearinghouse should not be considered a recommendation for implementation, because included strategies are sometimes ineffective. Second, little is known about strategies that are not listed in evidence clearinghouses. Are they ineffective, or have they simply not been studied or reviewed for inclusion?

## Guidance for Public Health Practitioners, Community Members, and Policy Makers

What knowledge do community leaders and policy makers need to be informed consumers of evidence clearinghouses that summarize evidence about health improvement efforts? As demonstrated in our qualitative review of publicly available data and in a 2016 assessment of education-related evidence resources, “the methods used in these syntheses vary in fundamental ways” (20). In using any evidence clearinghouse, paying attention to the fine print is important. Each clearinghouse has a unique approach to assessing evidence and communicating effectiveness. Particularly, the top evidence rating for some clearinghouses — communicating strategies that are most effective — requires a less thorough literature search with less robust results in some clearinghouses than others. This variability reflects different choices in search methods, replication requirements, and often, the scope of strategies included. Users of such clearinghouses can consult our list of key lessons as they examine the criteria of each clearinghouse to ensure that they understand the ratings and confirm ratings align with their local expectations and goals. Going forward, evaluation is needed to ensure that selected strategies work in the local population, setting, and context, as well as to add new examples to the evidence base.

Caution should be taken in implementing strategies that are found to have no effect or mixed results; communities interested in such strategies should consider study results, possible modifications to the strategy, and implications of implementation fidelity. Strategies for which literature reviews yield no qualifying studies might simply be too new to determine likely effectiveness. In these situations, conducting a pilot or implementing a rigorous evaluation to be sure that these strategies do, in fact, achieve expected outcomes is a wise approach.

Finally, evidence clearly matters to decision making but so do other factors. Knowledge building is a continuous process, and the creativity of local communities in addressing perplexing challenges, accompanied by a “test and see” approach, is often a source of new evidence. Local culture, potential effect on disparities, feasibility, and cost, are also important considerations. Purposeful approaches to balance these factors, along with evidence of effectiveness, can best support efforts to select strategies that will appropriately address local priorities.

## Post-Test Information

To obtain credit, you should first read the journal article. After reading the article, you should be able to answer the following, related, multiple-choice questions. To complete the questions (with a minimum 75% passing score) and earn continuing medical education (CME) credit, please go to http://www.medscape.org/journal/pcd. Credit cannot be obtained for tests completed on paper, although you may use the worksheet below to keep a record of your answers.

You must be a registered user on http://www.medscape.org. If you are not registered on http://www.medscape.org, please click on the “Register” link on the right hand side of the website.

Only one answer is correct for each question. Once you successfully answer all post-test questions, you will be able to view and/or print your certificate. For questions regarding this activity, contact the accredited provider, CME@medscape.net. For technical assistance, contact CME@medscape.net. American Medical Association’s Physician’s Recognition Award (AMA PRA) credits are accepted in the US as evidence of participation in CME activities. For further information on this award, please go to https://www.ama-assn.org. The AMA has determined that physicians not licensed in the US who participate in this CME activity are eligible for AMA PRA Category 1 Credits™. Through agreements that the AMA has made with agencies in some countries, AMA PRA credit may be acceptable as evidence of participation in CME activities. If you are not licensed in the US, please complete the questions online, print the AMA PRA CME credit certificate, and present it to your national medical association for review.

## Post-Test Questions

### Study Title: How to Be an Informed Consumer of Evidence Ratings: It’s in the Details

#### CME Questions

1. You are advising a public health department regarding informed decision making about potential strategies for health care policy implementation. According to the report and review by Bergum and colleagues, which of the following statements about evidence ratings by evidence clearinghouses is correct?
  - Evidence clearinghouses all use uniform evidence rating classifications and criteria for assigning ratings
  - All clearinghouses only report on strategies deemed to be “evidence-based”
  - What Works for Health (WWFH), the Healthy Communities Institute, and The Community Guide address multiple determinants of health; the latter two also address some specific diseases and injuries
  - All clearinghouses assign unfavorable as well as favorable ratings, and most have specific ratings for Expert Opinion
2. According to the report and review by Bergum and colleagues, which of the following statements about lessons learned from reviewing a sample of clearinghouses’ evidence of effectiveness ratings is correct?
  - All clearinghouses reviewed clearly define the types of strategies included (policies, programs, practices, or systems and environmental change)
  - The term “evidence-based” is widely used but has confusing and inconsistent definitions and standards across organizations and disciplines
  - All clearinghouses give greater weight to evidence from systematic reviews and randomized clinical trials (RCTs) than from other study types, particularly for highest evidence ratings
  - Requirements for literature review and considerations of study quality and quantity are standard across clearinghouses
3. According to the report and review by Bergum and colleagues, which of the following statements about guidance needed by public health practitioners, community leaders, and policy makers about evidence clearinghouses is correct?
  - For strategy selection and funding decisions, communities should balance evidence from clearinghouses with local needs, resources, and culture
  - Decision making should be based solely on currently available evidence
  - Strategies for which literature reviews yield no qualifying studies should not be tested further
  - Strategies shown to have no effect or mixed results should not be implemented
